# Impact of the best possible self intervention on affective well-being in early adolescence: A randomized controlled online trial

**DOI:** 10.1016/j.invent.2025.100827

**Published:** 2025-04-08

**Authors:** Stefanie Bartha, Silke Schmidt, Samuel Tomczyk

**Affiliations:** Department of Health and Prevention, University of Greifswald, Greifswald, Germany; German Center for Child and Adolescent Health (DZKJ), Partner site Greifswald/Rostock, Robert-Blum-Str. 13, 17489 Greifswald, Germany

**Keywords:** Positive psychology, Best possible self, Early adolescence, Affect, Randomized controlled trial, Online intervention

## Abstract

The Best Possible Self intervention (BPS) has demonstrated efficacy in promoting well-being in various populations, yet its impact in adolescence is under-researched. Our study investigated the feasibility and efficacy of the BPS in early adolescence (11–15 years) to promote positive affect and reduce negative affect. We conducted a randomized controlled online trial (*N* = 200, *M*_*age*_ = 14.01 years, *SD* = 1.19, 78.5 % female). Participants were assigned to the BPS group (*n* = 59), a writing control group (*n* = 68), or a non-writing control group (*n* = 73). Affect (PANAS-C-SF) was measured immediately before and after the intervention. The BPS demonstrated feasibility in our sample and significantly increased positive affect post-intervention compared to both control groups, suggesting a mood-boosting effect. The BPS did not significantly reduce negative affect post-intervention relative to the control groups. Our findings provide initial evidence that the BPS is a feasible and effective intervention for enhancing positive affect in early adolescence. Future research should explore its long-term effects, repeated administration, and potential for implementation in group settings to maximize its impact.

## Highlights

• First study to evaluate Best Possible Self intervention in early adolescence.
• BPS showed high feasibility with 84 % completion and 97 % adherence rates.
• BPS led to a significant increase in positive affect compared to both control groups.
• Repeated BPS intervention suggested for future studies to enhance efficacy.

## 1 Introduction

Adolescence is a period of intense physical, cognitive, and socio-emotional change, when decisions are made and habits are formed that can often have lifelong effects (Casey et al., 2008; Catalano et al., 2012; Daw et al., 2017; Steinberg, 2005). It is characterized by the increased ability for abstract thought and a search for a sense of identity (Sawyer et al., 2012). Thus, processes informing identity, habits, and goals during adolescence can affect life trajectories. For instance, developmental theories of delinquency, violence, and extremism, describe developmental processes (e.g., social identity, ideology) starting in childhood and adolescence as the root of these outcomes (e.g., Beelmann, 2021). Reversely, theories of positive development that are prevalent in positive psychological literature, such as the broaden-and-build theory, assume that early and repeated experiences of positive feelings, behaviors, and cognitions; such as hope for one's future, lead to positive self-perceptions and higher resilience and well-being (Ciocanel et al., 2017; Sanders et al., 2015; Schmid et al., 2011; Stifter et al., 2019). In this sense, interventions that focus on positive experiences and encourage the creation of positive goals and visions might positively influence developmental trajectories.

The Best Possible Self intervention (BPS), a future-oriented positive-psychological writing intervention, has demonstrated efficacy in promoting affective well-being in adult as well as young adult samples (Carrillo et al., 2019; Heekerens and Eid, 2021; King, 2001; Loveday et al., 2016; Malouff and Schutte, 2017; Schubert et al., 2020). However, the reviewed studies focus on adult populations, and so far, no studies have examined the impact of the BPS in adolescent samples as a stand-alone intervention. As adolescence represents a critical period for identity formation, the BPS, which encourages adolescents to envision their ideal future selves, may be particularly beneficial in facilitating this developmental process. Furthermore, empirical evidence suggests that a future-oriented perspective and hopefulness about one's future are significant predictors of positive youth development and are associated with improved health outcomes (Borowsky et al., 2009; Lindstrom Johnson et al., 2014; Seginer, 2009; Steinberg, 2008).

The BPS shows promise as a feasible and effective approach for enhancing well-being in young adolescents. As a positive psychology intervention, the BPS fits within the broader framework of interventions shown to enhance mental health in school settings (Tejada-Gallardo et al., 2020). Writing-based interventions in general have also shown to produce small but meaningful well-being improvements in adolescents (Travagin et al., 2015). Furthermore, Cohen et al. (2024) found that brief interventions significantly improve mental health outcomes in students, supporting the potential efficacy of a single-session BPS. Additionally, digital adaptations of mental health interventions have demonstrated efficacy (Fischer-Grote et al., 2024; Zhou et al., 2021), with web-based positive psychology exercises showing good adherence among adolescents with depression (Kaubisch et al., 2023). This suggests that the BPS could be successfully implemented in digital formats to increase accessibility. Overall, existing literature supports the BPS as a promising tool for improving adolescent well-being, though further research is needed to confirm its efficacy in this population. It is therefore highly advisable to investigate the feasibility and efficacy of the BPS in early adolescence.

The aim of the present study was to examine the feasibility of the BPS and its effect on positive and negative affect in early adolescence (11–15 years). Based on existing literature (Carrillo et al., 2019; Dimitropoulou and Leontopoulou, 2017; Heekerens and Eid, 2021; Schubert et al., 2020; Suldo et al., 2014), we propose the following hypotheses:

H1 The BPS intervention will demonstrate feasibility among early adolescents aged 11–15 years, as evidenced by high intervention completion rates, adherence to the instructions and an age-appropriate volume of text generated.

H2 The increase in positive affect is higher in the BPS group than in both control groups.

H3 The decrease in negative affect is stronger in the BPS group than in both control groups.

## 2 Materials and methods

### 2.1 Participants

**Fig. 1:**
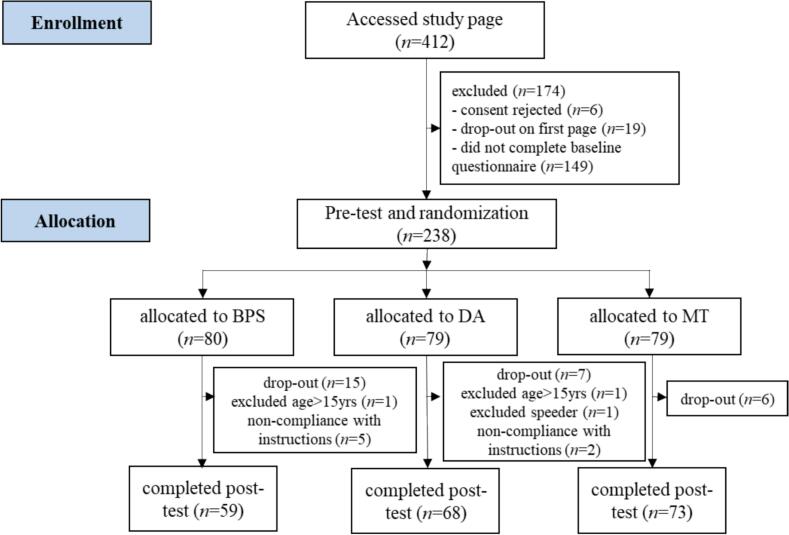
Flow diagram of progress of participants through the phases of the randomized controlled trial BPS = Best Possible Self (intervention group); DA = Daily Activities (writing control group); MT = Matrices Tests (non-writing control group).

Participants were recruited online through advertisements and posts on Instagram and Facebook, and e-mail distribution lists for parents. Recruitment was limited to Germany and German-speaking participants. In addition, posters and flyers were distributed in youth centers, and pediatric practices in Western Pomerania. As an incentive, all participants had the opportunity to take part in a prize draw for gift vouchers. At the end of the online questionnaire, the young people were asked whether they would like to take part in the prize draw for a total of 20 gift vouchers worth 15 euros each. After the end of data collection, the vouchers were then distributed at random among all participants who answered in the affirmative. The winners were subsequently sent the voucher codes by e-mail. To determine the required sample size, we conducted an a priori power analysis. With an estimated effect size on positive affect of Hedge's *g* = 0.28 (Heekerens and Eid, 2021), a significance level of α = 0.05 and a power of 1-β = 0.80, the sample size for a comparison of three groups was 126 participants. We included adolescents who were at least 11 and not more than 15 years old. The sample at the time of intervention consisted of 200 adolescents, 59 in the BPS condition, 68 in the writing control group (Daily Activities; DA) and 73 in the non-writing control group (Matrices Tests; MT) condition. The mean age was 14.01 years (*SD* = 1.19), 78.5 % were female, and 1.5 % indicated a gender other than male or female (see Table 1 for the distribution of age and gender by group). Data were collected from December 2020 to March 2021. Participant flow is documented in Fig. 1.

**Table 1:** Descriptive statistics on age, gender and the outcome variables before (t0) and after intervention (t1) for the total sample and by group.

		Total sample (*n* = 200)			Best Possible Self (*n* = 59)			Daily Activities (*n* = 68)			Matrices Tests (*n* = 73)		
Variable		*N*	*M*	*SD*	*N*	*M*	*SD*	*N*	*M*	*SD*	*N*	*M*	*SD*
Age		200	14.01	1.19	59	13.85	1.16	68	14.00	1.27	73	14.15	1.13
Gender (% female)		200	78.5		59	78.0		68	76.5		73	80.8	
Positive Affect	t0	200	2.87	0.95	59	2.90	0.88	68	2.87	0.95	73	2.84	1.01
	t1	200	2.87	1.00	59	3.02	0.92	68	2.81	0.99	73	2.80	1.08
Negative Affect	t0	200	2.03	0.83	59	1.94	0.68	68	2.09	0.90	73	2.05	0.87
	t1	200	1.80	0.78	59	1.66	0.65	68	1.88	0.86	73	1.84	0.81

### 2.2 Procedure

Participants were informed about the aim of the study, the voluntary nature of participation in the study and data protection. After obtaining participants' and their legal guardians' consent, the participants were randomly assigned to either the intervention group (BPS) or one of the two control groups (DA and MT) using the “random trigger” function provided by the Unipark survey platform. To ensure balanced group distribution, the Unipark feature “aim for equal distribution in specified area” was enabled. This function progressively limits available options - it is a random draw without replacement - approximating uniform distribution even with small sample sizes. We employed two control groups, writing about daily activities (i.e., writing control group, DA), and performing problem-solving tasks (i.e., non-writing control group, MT; Weiß, 1987). We found this necessary in order to discern effects of writing per se from the effects of writing about an ideal future, since even neutral writing can have positive effects compared to, for example, a waiting list group (Gerger et al., 2021). Participants in the BPS condition were instructed to write about their ideal future for 10 min, participants in the DA condition were instructed to write in detail about their previous day for 10 min and participants in the MT condition were asked to continue a row of figures by choosing one of five figures that best matched a row of three figures (Weiß, 1987). They also had a total of 10 min to complete the tasks. Before the randomized controlled online trial, we held six focus groups with 22 young adolescents (*N* = 22, *M*_*age*_ = 12.48 years, *SD* = 1.7, age range = 9–15 years) from 5 youth centers to assess study feasibility and acceptance. Analysis of these groups led to shortening the intervention from the original 20 (King, 2001) to 10 min. Participants in the writing conditions were informed that their texts are confidential, that neither teachers nor parents will read their texts and that their texts will not be evaluated or graded. All instructions were given in German.

All participants completed an online questionnaire on personal and social resources (such as optimism, self-efficacy and parental support), affect, life satisfaction and depressive symptoms before randomization and pre-intervention. Positive and negative affect were measured again immediately after the intervention. After that, adolescents were asked to fill out a questionnaire on socio-demographic data. Ethical approval was granted from the Ethics Committee of the University Medicine Greifswald (reference number: BB 078/19).

### 2.3 Measures

#### 2.3.1 Feasibility

Feasibility was assessed in terms of intervention completion, adherence to the instructions and the volume of the generated texts. Intervention completion refers to the proportion of participants who remain engaged throughout the entire duration of the intervention. High completion rates indicate the intervention's acceptability and practicality among the target population. This measure of feasibility is employed in studies evaluating both online and offline psychosocial interventions (Corn et al., 2023; Fallon et al., 2024; Morgan et al., 2008; Murray et al., 2022; Nguyen et al., 2020). The intervention completion rate was calculated as the number of participants who completed the post-intervention assessment (post-test) out of the total participants who completed the pre-intervention assessment (pre-test). Adherence measures how closely participants follow the prescribed procedures or guidelines of the intervention (Hanano et al., 2022). High adherence rates suggest that participants find the intervention manageable and relevant and are engaging with the intervention as intended, which supports the practicality and potential effectiveness of the program. To measure adherence, two independent coders (first and senior author) went through the texts to check whether youth had understood the instructions correctly and executed them accordingly. BPS texts were counted as valid when youth wrote about their future in a positive way. DA texts were counted as valid when youth wrote about their previous day in any form. MT answers were checked for missing values and response sets. In addition, we also used the length of the texts as a descriptive indicator of the feasibility of the intervention. In interventions that involve participants generating textual content, the volume of these texts serves as an indicator of engagement and the intervention's acceptability. A higher volume of generated texts may reflect participants' willingness to engage deeply with the intervention material. The average number of sentences written and the average number of words written were determined in order to measure the volume of text generated. Young adolescents typically produce between 9 and 22 words per minute in narrative writing tasks. Therefore, in a 10-min period, they could generate approximately 90 to 220 words (Atasoy and Temizkan, 2016; Olive et al., 2009). We compared the number of words generated in the texts with the standard that was collected in previous studies on narrative writing.

#### 2.3.2 Affect

Affect was assessed using the Positive and Negative Affect Schedule for Children – German version (Ebesutani et al., 2012; Laurent et al., 1999; Weber, 2020). The PANAS-C-SF is a 10-item self-report scale measuring positive and negative affect in children and adolescents. The original version asks participants to rate their affect in general on a 5-point scale ranging from ‘not at all’ (1) to ‘extremely’ (5). As we were interested in short-term changes in affect, we rephrased the wording of the instructions from ‘in general’ to ‘at the moment’. Example items include ‘sad’, ‘happy’, ‘unhappy’ and ‘afraid’. The test showed good to acceptable internal consistency with α = 0.90 for the subscale *positive affect* and α = 0.80 for the subscale *negative affect*.

### 2.4 Statistical analyses

To assess the stability of individual differences in affect over time, we computed test-retest correlations for positive and negative affect between pre- and post-intervention in each of the three groups (BPS, DA, MT) using Pearson's correlation coefficient. Higher values indicate greater stability of individual differences across time. To examine whether these retest correlations differed significantly between groups, Fisher's z-tests were used (Fisher, 1970). One-sided tests were employed to test the directional hypothesis that stability may be weaker in the BPS group. Comparisons included BPS vs. DA, BPS vs. MT, and DA vs. MT for both affect measures. *P*-values were adjusted for multiple comparisons using the Benjamini-Hochberg correction (*q* = 0.05; Benjamini and Hochberg, 1995).

To test our hypothesis that the increase in positive affect is higher in the BPS group than in both control groups, we conducted two one-sided *t*-tests comparing the mean change scores between the BPS group and the DA or the MT group, respectively. We did the same for our hypothesis that the decrease in negative affect is stronger in the BPS group than in both control groups. To address the issue of the false discovery rate (FDR) due to multiple testing, we applied the Benjamini-Hochberg correction with *q* = 0.05 to both sets of statistical tests (Benjamini and Hochberg, 1995). We used IBM SPSS Statistics Premium (versions 28 and 29) for all our analyses.

## 3 Results

### 3.1 Preliminary analyses

Prior to the main analysis, we conducted a MANOVA to test whether participants in the BPS, DA and MT groups differed in their baseline scores on positive and negative affect and age. The analysis showed no statistically significant difference in any of the dependent variables, *F*(6, 392) = 0.514, *p* = .798, partial η^2^ = 0.008, Pillai's Trace = 0.016. A χ^2^-test for association was conducted between gender, and group. Three participants who indicated a gender other than male or female were excluded from this analysis due to their small number. Results show no significant differences between gender, and group, χ^2^(2) = 0.273, *p* = .873, φ = 0.037. In sum, there were no significant differences between the three conditions at baseline. The descriptive statistics of outcome variables by group at pre-, and post-intervention are displayed in Table 1. One participant had to be excluded for speeding because his response time was less than half of the median response time for completing baseline assessment (Jandura, 2017).

We also calculated the test-retest correlations across experimental groups to examine interpersonal changes across time, which resulted in *r* = 0.802 for positive affect in the BPS group, *r* = 0.861 in the DA group, and *r* = 0.906 in the MT group (all *p* < .001). For negative affect, correlations were also large with *r* = 0.735 in the BPS group, *r* = 0.870 in the DA group, and *r* = 0.849 in the MT group (all *p* < .001). One-sided Fisher's z-tests revealed significantly lower retest correlations in the BPS group compared to MT for positive affect (*z* = −2.23, *p* = .013), and compared to both DA (*z* = −2.16, *p* = .015) and MT (*z* = −1.75, *p* = .040) for negative affect. The differences remained significant after applying the Benjamini-Hochberg correction. These results suggest greater variability of interindividual differences over time in the BPS group compared to DA and MT, which aligns with our hypothesis that the BPS has an affect modifying influence. There were no statistically significant differences between the retest correlations of the BPS group compared with the DA group for positive affect and between the two control groups for both positive and negative affect.

### 3.2 Feasibility

#### 3.2.1 Intervention completion

Of the 238 participants who completed the pre-test assessment and were subsequently randomized, 200 completed both the intervention and the post-test assessment, resulting in an overall intervention completion rate of 84.03 %. Within the different groups, 59 out of 80 participants in the BPS group completed the post-test, 68 out of 79 in the DA group and 73 out of 79 in the MT group. This corresponds to completion rates for the individual groups of 73.75 %, 86.08 % and 92.41 % for the BPS, DA, and MT groups, respectively.

#### 3.2.2 Adherence to the instructions

In the BPS group, three participants did not write any text at all, one person merely repeated the instructions, and another created a negative picture of their future. In total, five participants had to be excluded from the BPS group. In the DA group, two adolescents did not write about their past day and were therefore excluded. In the MT group, there were no missing values and no response sets; all MT participants were included in the analyses. In total, 200 out of 207 participants adhered the instructions as intended, resulting in an adherence rate of 96.62 %.

#### 3.2.3 Volume of the generated texts

The final sample of BPS texts generated by the youth consisted of 59 texts. The average number of sentences written by participants was 12.41 (*SD* = 8.09), with a minimum of 1 and a maximum of 37 sentences. The average number of words written by participants was 186.76 (*SD* = 109.59), with 4 participants out of 59 (6.78 %) producing fewer than 50 words and a total of 13 participants (22.03 %) producing less than 90 words, the lower limit of the average words produced narratively in the age group (Atasoy and Temizkan, 2016; Olive et al., 2009). The average number of words produced in the BPS texts thus falls within the range of 90–220 words typically produced by young adolescents in 10 min (see above, Atasoy and Temizkan, 2016; Olive et al., 2009). There was a statistically significant difference between the number of sentences and words generated by female and male participants, with an average of 9.45 sentences and 74.74 words fewer in the male group, *t*(55) = 3.87, *p* < .001 and *t*(55) = 2.07, *p* = .043, respectively. There was a small positive correlation between age and the number of sentences and words generated, *r* = 0.158, *p* = .233 and *r* = 0.231, *p* = .079, respectively.

### 3.3 Main analyses

**Fig. 2:**
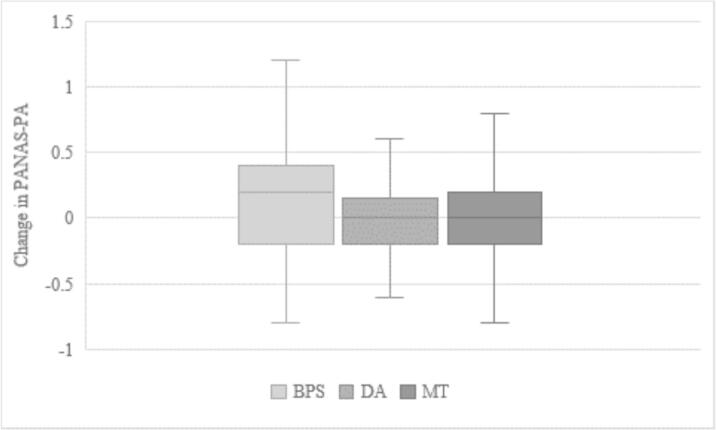
Boxplot for change in positive affect by group (*n* = 200) PANAS-PA = Positive and Negative Affect Schedule, Subscale Positive Affect; BPS = Best Possible Self (intervention group); DA = Daily Activities (writing control group); MT = Matrices Tests (non-writing control group).

There was a statistically significant difference regarding change in positive affect between the BPS group and the two control groups, *t*(125) = 1.86, *p* = .033 for the DA group and *t*(130) = 1.83, *p* = .035 for the MT group. Both comparisons remained statistically significant after applying the Benjamini-Hochberg correction (with *p* = .035 below the critical value of 0.05) (Fig. 2).

**Fig. 3:**
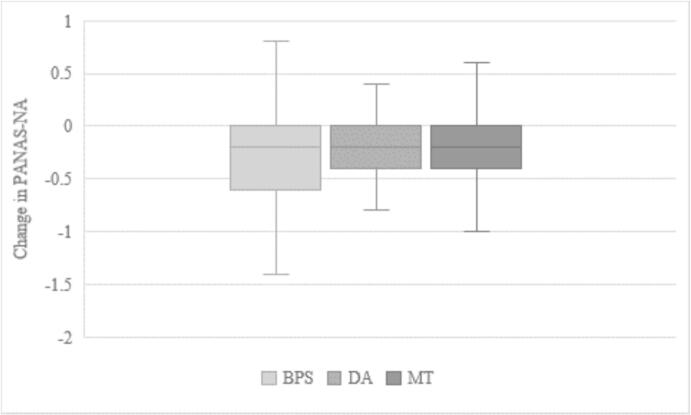
Boxplot for change in negative affect by group (*n* = 200) PANAS-NA = Positive and Negative Affect Schedule, Subscale Negative Affect; BPS = Best Possible Self (intervention group); DA = Daily Activities (writing control group); MT = Matrices Tests (non-writing control group).

There was no statistically significant difference regarding the change in negative affect between the BPS group and the two control groups, *t*(125) = −0.87, *p* = .192 for the DA group and *t*(130) = −0.85, *p* = .199. Both comparisons remained non-significant after applying the Benjamini-Hochberg correction (both *p*-values above their respective critical value of 0.025 and 0.050) (Fig. 3).

## 4 Discussion

The aim of our study was to examine the feasibility and the efficacy of the Best Possible Self intervention (BPS) to promote positive affect and to decrease negative affect in early adolescence (11–15 years). In our study, the BPS has shown to be a feasible intervention for young adolescents. The total intervention completion rate was 84.03 %. The majority of our participants adhered to the instructions (96.62 %) and generated a number of words (on average 186.76 words) that falls within the range of the number of words typically produced by young adolescents in narrative writing.

The results of our randomized controlled trial indicate that the BPS significantly increased positive affect post-intervention compared to two control groups (writing about daily activities, performing a problem-solving task), indicating a mood-boosting tendency of the BPS. The BPS had no significant effect on negative affect post-intervention compared to the two control groups. These findings are in line with recent meta-analyses on the BPS in adult populations who found the BPS to have a positive affect-inducing effect and a non-significant or small effect on negative affect (Carrillo et al., 2019; Heekerens and Eid, 2021; Schubert et al., 2020).

### 4.1 Feasibility and practical implications

Our study demonstrates the feasibility of the Best Possible Self (BPS) intervention for early adolescents, as evidenced by high intervention completion rates, adherence to instructions, and an average word count consistent with typical adolescent writing (Atasoy and Temizkan, 2016; Olive et al., 2009). These findings suggest that young adolescents are capable of engaging with future-oriented, self-reflective tasks, aligning with theories of adolescent cognitive development, which posit an increasing capacity for abstract thought and self-reflection during this period (Sawyer et al., 2012). Our results support the feasibility of short, scalable interventions, aligning with research demonstrating that even brief psychological interventions can produce meaningful benefits (Cohen et al., 2024). Moreover, the feasibility of a digital format highlights its potential for increasing accessibility, engagement, and personalization, supporting the broader shift toward web-based mental health interventions (Fischer-Grote et al., 2024; Zhou et al., 2021). The feasibility of the BPS in this age group highlights the potential of brief positive psychology interventions within, for example, school-based mental health programs, supporting previous findings on their benefits (Tejada-Gallardo et al., 2020).

### 4.2 Effects on positive and negative affect

Consistent with prior research on adult populations, the BPS intervention significantly increased positive affect post-intervention but did not significantly reduce negative affect (Carrillo et al., 2019; Heekerens and Eid, 2021; Schubert et al., 2020). The observed increase in positive affect is particularly relevant, as emotional well-being and positive affect in adolescence are linked to long-term benefits, including improved academic performance, social outcomes, health behaviors, and mental health (Kim et al., 2024; Marrone et al., 2024). This aligns with the broaden-and-build theory (Fredrickson, 2001), which posits that positive emotions expand cognitive and behavioural repertoires, fostering resilience and adaptive functioning. Given that adolescence is a formative period for identity development and goal formation (Sawyer et al., 2012), the BPS may serve as a valuable tool in fostering an optimistic and agentic self-view and potentially contribute to the promotion of positive youth development and resilience-building. Since the BPS did not lead to reduction in negative affect, we assume that the BPS is useful to foster positive emotions but not alleviate negative ones, which aligns with some definitions of positive psychological interventions (Carrillo et al., 2019). Notably, in our sample, negative affect decreased across all three groups post-test (see Table 1). This suggests that participation in the study, regardless of the intervention content, may have had a positive impact on negative affect. One possible explanation could be that especially in pandemic times, at the time of the study, adolescents were experiencing negative affect daily, connected to pessimistic media reports, uncertainty regarding the future, and a lack of opportunity for positive divertisement, due to school lockdown policies and limited social interaction. In this situation, a structured, self-administered activity such as diary writing or solving puzzles might be a welcome distraction and divert attention from negative affect to more neutral or even positive tasks. In a pre-pandemic study by Woodworth et al. (2017), three web-based positive-psychological interventions as well as the placebo intervention lead to significant increases in happiness and reductions in depression. They attributed these effects to non-specific factors common to most treatments. A detailed investigation of factors common to web-based intervention studies' arms could be beneficial. Noticeably, though, we found group differences in positive affect, suggesting differential impact of BPS instructions beyond these non-specific factors.

### 4.3 Future research directions

We measured the immediate effect of the BPS on affect, future studies should incorporate follow-up assessments to determine whether the BPS intervention leads to sustained benefits. Future research could also explore the impact of repeated BPS exercises over time to determine whether multiple sessions enhance its effectiveness. Prior studies suggest that positive psychological interventions yield stronger effects when implemented over extended periods, as they allow participants to internalize and transform positive activities into habits (Bolier et al., 2013; Carr et al., 2021; Sin and Lyubomirsky, 2009). Given that the original 20-min intervention duration (King, 2001) led to frustration and fatigue among participants from the pilot phase in our study, a shorter yet more frequent implementation - such as administering the BPS four times at one-week intervals (cf. Layous et al., 2013) - could be a promising alternative. However, findings on the role of intervention duration remain inconclusive, as some studies report no significant effects or even a negative association between duration and effect size (Carrillo et al., 2019; Tomczyk et al., 2023). Consequently, empirical research examining both the optimal duration and the potential benefits of repeated BPS exercises is needed to refine its implementation in adolescent populations. Future research could explore additional outcomes that have been previously linked to the BPS intervention in adults, such as optimism and other well-being indicators (Carrillo et al., 2019; Heekerens and Eid, 2021). Furthermore, it would be valuable to examine outcomes particularly relevant to adolescence, such as identity formation (Branje et al., 2021), to better understand the broader impact of the intervention in this developmental stage. In addition, while we controlled for general writing effects, comparing the BPS to other positive psychology interventions, such as gratitude exercises, could clarify its relative efficacy (Owens and Patterson, 2013; Peters et al., 2013). Exploring combinations of interventions may also be beneficial for addressing both positive and negative affect. Finally, while the overall intervention completion rate was high (84.03 %), notable discrepancies were observed across the three conditions, with the BPS group showing the lowest retention (73.75 %) compared to the DA (86.08 %) and MT (92.41 %) groups. This may be partially explained by the emotional and cognitive demands associated with imagining and articulating one's best possible self. Prior research suggests that expressive writing interventions can be perceived as effortful or emotionally taxing, particularly for individuals low in emotional expressiveness (Niles et al., 2014). This may be especially relevant for adolescents, who are still developing cognitive, emotional, and narrative processing capacities (Fivush et al., 2007). Travagin et al. (2015) propose that higher dropout rates may reflect limited psychosocial resources or underdeveloped emotional regulation skills. The MT group, which involved no writing, likely had lower attrition due to reduced cognitive and emotional demands. Future research could explore ways to enhance engagement and retention in writing-based interventions, such as adjusting task length, providing structured guidance, or integrating motivational support (Layous et al., 2013). Additionally, investigating participant characteristics associated with intervention completion could provide insights into tailoring interventions to better match individuals' needs and preferences (Lyubomirsky and Layous, 2013).

### 4.4 Limitations

While our study provides initial evidence for the feasibility and efficacy of the BPS intervention, several limitations should be noted. First, our sample is a self-selected sample recruited primarily through social media in German-speaking countries and consists mostly of female participants (77.8 %) who are pursuing a more academically rigorous educational track. Specifically, 57.5 % of participants attend a ‘Gymnasium’, which is the most academically oriented type of secondary school in the German education system. Therefore, our findings may not apply to other subgroups of the adolescent population. Additionally, we relied exclusively on self-report measures of the outcome variables and the battery of questionnaires was relatively lengthy (about 30 min at baseline). Although we specifically used instruments for adolescents or children, the length of the battery of questionnaires may have led to cognitive and emotional fatigue effects that influenced affect alongside the intervention (Midgley et al., 2016).

### 4.5 Conclusion

Our study provides initial evidence that the BPS intervention is a feasible and effective tool for enhancing positive affect in early adolescence. These findings contribute to the growing field of positive psychology interventions for youth and highlight the potential of future-oriented interventions in supporting adolescent well-being. As adolescence is a critical period for identity formation and the development of future perspectives, interventions like the BPS may play a crucial role in fostering positive developmental trajectories. Future research should explore long-term effects, and the impact of repeated administration in diverse adolescent populations. Implementing the BPS in group settings such as classrooms or youth programs could further enhance its accessibility and impact.

## Funding

This work was supported by the Landesgraduiertenförderung Mecklenburg-Vorpommern (State Graduate Funding Mecklenburg-Western Pomerania, Bogislaw Scholarship) at the University of Greifswald, Germany. Support for the publication fee was provided by University of Greifswald's publication fund.

## Declaration of competing interest

The authors declare that they have no known competing financial interests or personal relationships that could have appeared to influence the work reported in this paper.

## Data Availability

The data that support the findings of this study are available on reasonable request from the corresponding author.
